# AG1® supplementation improves gut microbiome composition and function in highly active adults

**DOI:** 10.1080/15502783.2025.2550154

**Published:** 2025-10-06

**Authors:** Jeremy Townsend, Adam M. Gonzalez, Trevor Kirby, Philip Sapp, Caitlyn Edwards, Justin Wright, Gina Lamendella, Lara Nyman, Ralph Esposito

**Affiliations:** a Research, Nutrition, and Innovation, AG1, Carson City, NV, USA; b Concordia University Chicago, Health & Human Performance, River Forest, IL, USA; c Hofstra University, Department of Allied Health and Kinesiology, Hempstead, NY, USA; d Wright Labs, Huntingdon, PA, USA; e New York University-Steinhardt, Department of Nutrition, Food Studies and Public Health, New York, NY, USA

**Keywords:** Active, gut health, supplement

## Abstract

**Background:**

The objective of our study was to explore how 2 weeks of AG1_®_ supplementation influence the structure and metagenomic function of the gut microbiome in highly active men and women.

**Methods:**

A double-blind, randomized, placebo-controlled crossover designed study was conducted in 20 highly active male (*n* = 10; 26.4 y; 85.6 kg) and female (*n* = 10; 26.9 y; 70.0 kg) adults with an average of 9.7 years of resistance training experience. Each participant maintained their normal diet and supplemented with AG1_®_ (13 g/day) and placebo (maltodextrin matched for color and taste; 13 g/day) in a randomized, counterbalanced order for 14 days with a 2-week washout period in-between treatments. Stool was collected and placed in DNA/RNA Shield (Zymo Research, USA) from each participant before and after each trial period to assess changes in the gut microbiome. DNA was extracted and subsequent libraries were prepared for whole genome shotgun metagenomic sequencing at ~8 M reads sequence depth. Quality control steps were conducted at each step. Sequences were annotated using Kraken2 to identify the taxa present. Functional annotations were performed using Emapper v2.0 and the eggNOG 5.0 database to determine which KEGG orthologs were present. The structural and functional annotation steps were normalized to counts per million.

**Results:**

No significant differences were detected in the number of observed species or Pielou’s Evenness between the two treatment groups. There were no significant differences in overall community composition as indicated by PERMANOVA analysis (*p* > 0.05). Despite this, there was clear clustering between the treatment groups at each time point using PLS-DA ordination. LEfSe analysis revealed that AG1 supplementation lead to significant (*p* ≤ 0.0001) enrichment of 6 taxa including Bifidobacterium animalis, Lacticaseibacillus rhamnosus, and Lactiplantibacillus plantarum. Additionally, the enrichment of various taxa caused several gene pathways to have greater representation within the microbiome implying potential metabolic benefits that could be conferred to the host. Further, in a subset of participants (*n* = 10), wash-out analysis revealed that these benefits were lost with an observed significant decrease (*p* ≤ 0.0001) in 11 taxa.

**Conclusions:**

AG1 supplementation significantly improved the abundance of beneficial taxa and community function of the gut microbiome in highly active males and females.

